# Arterial Hypertension: Novel Pharmacological Targets and Future Perspectives

**DOI:** 10.3390/jcm13195927

**Published:** 2024-10-04

**Authors:** Irene Paula Popa, Andreea Clim, Alin Constantin Pînzariu, Cristina Iuliana Lazăr, Ștefan Popa, Ivona Maria Tudorancea, Mihaela Moscalu, Dragomir N. Șerban, Ionela Lăcrămioara Șerban, Irina-Iuliana Costache-Enache, Ionuț Tudorancea

**Affiliations:** 1 Department of Morpho-Functional Sciences II, Discipline of Physiology, “Grigore T. Popa” University of Medicine and Pharmacy, 700115 Iași, Romaniadragomir.serban@umfiasi.ro (D.N.Ș.);; 2 2nd Department of Surgery–Pediatric Surgery and Orthopedics, “Grigore T. Popa” University of Medicine and Pharmacy, 700115 Iași, Romania; 3 Advanced Research and Development Center for Experimental Medicine (CEMEX), “Grigore T. Popa” University of Medicine and Pharmacy, 16 Universității Street, 700115 Iași, Romania; 4 Department of Preventive Medicine and Interdisciplinarity, “Grigore T. Popa” University of Medicine and Pharmacy, 700115 Iași, Romania; 5 Department of Internal Medicine I, Grigore T. Popa” University of Medicine and Pharmacy, 700115 Iași, Romania; ii.costache@yahoo.com; 6 Cardiology Clinic, “St. Spiridon” County Clinical Emergency Hospital, 700111 Iași, Romania

**Keywords:** hypertension, natriuretic peptide, neprilysin, endothelin receptor, NO pathway

## Abstract

Arterial hypertension (HTN) is one of the major global contributors to cardiovascular diseases and premature mortality, particularly due to its impact on vital organs and the coexistence of various comorbidities such as chronic renal disease, diabetes, cerebrovascular diseases, and obesity. Regardless of the accessibility of several well-established pharmacological treatments, the percentage of patients achieving adequate blood pressure (BP) control is still significantly lower than recommended levels. Therefore, the pharmacological and non-pharmacological management of HTN is currently the major focus of healthcare systems. Various strategies are being applied, such as the development of new pharmacological agents that target different underlying physiopathological mechanisms or associated comorbidities. Additionally, a novel group of interventional techniques has emerged in recent years, specifically for situations when blood pressure is not properly controlled despite the use of multiple antihypertensives in maximum doses or when patients are unable to tolerate or desire not to receive antihypertensive medications. Nonetheless, reducing the focus on antihypertensive medication development by the pharmaceutical industry and increasing recognition of ineffective HTN control due to poor drug adherence demands ongoing research into alternative approaches to treatment. The aim of this review is to summarize the potential novel pharmacological targets for the treatment of arterial hypertension as well as the future perspectives of the treatment strategy.

## 1. Introduction

Although arterial hypertension (HTN) is one of the most significant and preventable cardiovascular risk factors [1], it remains the leading cause of cerebrovascular (51%) and cardiovascular (45%) death worldwide [2]. Remarkable advances have been achieved in recent years for a comprehensive understanding of the pathophysiology of HTN. Epidemiological studies state that even a decrease of 10 mmHg in systolic blood pressure (SBP) levels significantly lowers the risk of major cardiovascular disease events by 20%, coronary heart disease by 17%, heart failure by 28%, stroke by 17%, and all-cause mortality by 13% [3]. Despite the availability of a plethora of antihypertensive drugs, the blood pressure (BP) control rate continues to be considerably below acceptable levels [4,5,6,7]. Therefore, the pharmacological and non-pharmacological management of HTN is currently the major focus of healthcare systems. In the present paper, we aim to outline pharmacological and nonpharmacological approaches as well as future perspectives for the treatment of arterial hypertension.

## 2. New Drug Targets in Arterial Hypertension

### 2.1. Natriuretic Peptide and Neprilysin

In addition to pumping blood, the heart serves as an endocrine system that releases hormones, including natriuretic peptides (NPs), which are currently known to be involved in the regulation of BP, cardiac structure, and water balance [8]. Atrial natriuretic peptide (ANP), B-type natriuretic peptide (BNP), and C-type natriuretic peptide (CNP) are some of the several NPs released by the human heart. These NPs function via receptors (NPRs, natriuretic peptide receptors) and are classified as NPR-A, NPR-B, and NPR-C [9]. These NPs bind with the membrane-bound particulate guanylyl cyclase (pGC), which exists in both a membrane-spanning (pGC) and soluble form (sGC) inside cells [10]. Targeting these natriuretic peptides and their subsequent pathways may be a promising approach for the treatment of HTN. For example, in a canine model of ischemia-induced acute renal dysfunction, the pGC-A activator/designed natriuretic peptide CRRL269 induced vasorelaxation and antihypertensive effects [11]. Another pGC-A activator, MANP (ZD100), was shown in a human study to be safe and very effective at lowering BP. Additionally, this pGC-A activator decreased the concentration of aldosterone and developed renal protection [12]. Furthermore, ANX042, an additional pGC-A activator that stimulates the secondary messenger cGMP, was also demonstrated to be safe and effective. When administrated, ANX042 induced significant diuretic and natriuretic effects but without vasodilatory hypotensive features [13].

Neprilysin (NEP), a membrane-bound zinc endopeptidase found in a variety of organs, has been shown to contribute to the breakdown of vasoactive peptides, including bradykinin and NPs. Therefore, a neprilysin inhibitor (NEPi) will impede the breakdown of vasoactive peptides such as bradykinin, calcitonin gene-related peptide, adrenomedullin, and endothelin-1 [14]. Vasodilation; improved diuretic effect; natriuresis; decreased sympathetic activity; and long-term promotion of anti-inflammatory, antifibrotic, and antihypertrophic actions on cardiomyocytes in vitro are among the positive effects of NEPi [15]. Considering the lack of safety and effectiveness evidence, therapy with NEPi alone could not compete with existing medications, and thus, it has prompted research into the effective, safe, and advantageous combinations of NEPi with angiotensin II Receptor Blockers (ARB)/angiotensin-converting enzyme inhibitors (ACE) classes. Suppression of NEP, in addition to renin–angiotensin–aldosterone system (RAAS) blockers, enhances the bioavailability of protective NPs and facilitates cardiorenal disease therapy [16]. Chronic dual NEP-endothelial converting enzyme (ECE) suppression was associated with a considerable improvement in endothelial function and renovascular protection [17]. Several cardiovascular benefits are associated with angiotensin receptor-neprilysin inhibitors (ARNI) [18]. LCZ696 (sacubitril/valsartan) was the first ARNI that showed BP-lowering effects [19]. In a randomized, double-blind, and placebo-controlled study that included 1328 patients with mild-to-moderate HTN, progressive doses of sacubitril/valsartan were examined in comparison to progressive doses of valsartan alone and to sacubitril alone [19]. When compared to valsartan alone, sacubitril/valsartan considerably reduced office systolic (SBP) and diastolic blood pressure (DBP) and 24-h ambulatory SBP. Interestingly, sacubitril alone decreased BP much more than a placebo but less than sacubitril/valsartan or valsartan alone [19]. Moreover, LCZ696 was also found to lower BP in highly hypertensive Asian patients (office BP > 180/110 mmHg) in a safe and well-tolerated manner [20]. The development of hypertensive heart disease is heavily influenced by the global hemodynamic impact on arterial stiffness and nocturnal BP. Consequently, these may be the mechanistic targets behind the impact of ARNI on the spectrum of cardiovascular illness, from hypertension to heart failure [18].

**Figure 1:**
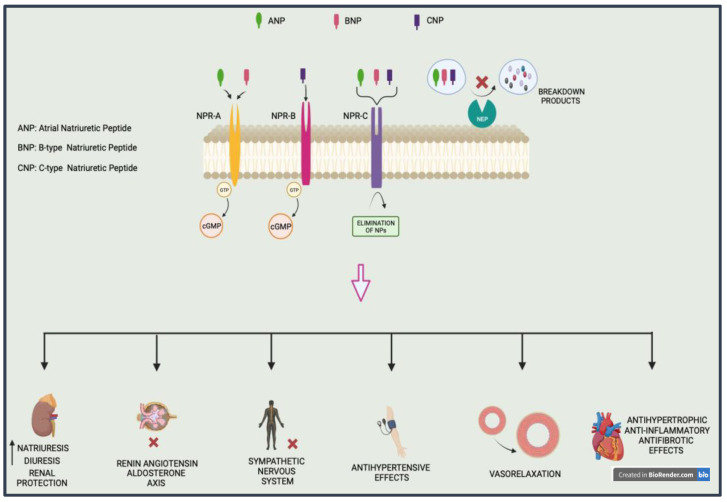
The effects of the modulation of the natriuretic peptides pathways on the cardiovascular system; ANP: atrial natriuretic peptide; BNP: B-type natriuretic peptide; CNP: C-type natriuretic peptide; NPR-A: natriuretic peptide receptor A; NPR-B: natriuretic peptide receptor B; NPR-C: natriuretic peptide receptor C; NEP: neprilysin; GTP: guanosine triphosphate; cGMP: cyclic guanosine monophosphate; 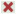: inhibition.

Other proposed mechanisms encompass decreases in circulating volume and sympathetic activity, both of which ensure the protection against target organ injury and favorable changes in cardiac biomarkers observed during ARNI treatment [18]. Mechanisms of action and positive outcomes of ARNI are supplementary to those of many other hypertension therapies, indicating the probability of cumulative or even synergistic effects [18]. Recent evidence suggests that patients with salt-sensitive hypertension, structural hypertension, resistant hypertension (RHTN), and hypertension in the context of heart failure would benefit the most from ARNI antihypertensive effects [18]. All the positive effects of the modulation of the natriuretic peptide pathways are summarized in Figure 1.

### 2.2. Stimulation of Soluble Guanylyl Cyclase A

**Figure 2:**
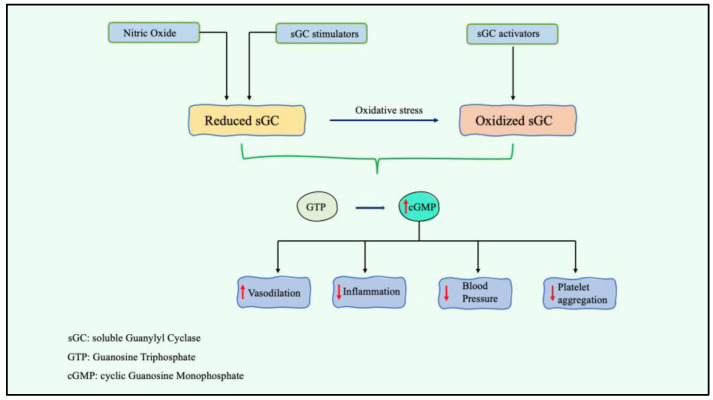
The role of the activation of soluble guanylyl cyclase on vasodilation, inflammation, blood pressure regulation and platelet aggregation; sGC: soluble guanylyl cyclase; GTP: guanosine triphosphate; cGMP: cyclic guanosine monophosphate.

Soluble guanylate cyclase (sGC) has an essential and well-established involvement in numerous (patho)-physiological mechanisms, especially in the cardiovascular system [21]. Therefore, sGC is a promising therapeutic focus in cardiovascular diseases such as myocardial infarction [22], myocardial dysfunction linked to sepsis [23], and stroke [24]. A substantial amount of research has emphasized the possible involvement of sGC in the onset of HTN [21]. sGC stimulators induce cardiorenal protection with the support of cardiac hormones and their endogenous ligands by attaching to sGC and stimulating the synthesis of cGMP [25]. The NO/sGC/cGMP signaling transduction pathway modulates several cardiac and renal disorders [26]. There are two major determinants: the first is the gaseous signaling molecule nitric oxide (NO), which is primarily accountable for vital functions such as vasorelaxation, cell proliferation, neurotransmission, immunity, platelet aggregation, and mitochondrial respiration. The downregulation of NO signaling results in cardiovascular disorders, renal dysfunction, sepsis, pulmonary damage, and failure of major organs [27]. The second and most significant determinant is oxidative stress, which disrupts the NO/sGC/cGMP pathway [8]. Studies employing genetically engineered animal models, synthetic stimulators, and inhibitors of the NO/sGC signaling system as well as genetic association studies in humans have underlined the importance of sGC in BP regulation. Several studies have highlighted NO/sGC’s contribution to vasodilation and vascular dysfunction, and thus, sGC stimulators may become a popular therapy option for cardiovascular and renal conditions [21]. sGC stimulators also have potential antifibrotic, antihypertrophic, and anti-inflammatory properties [28]. In an experimental model of cardiorenal insufficiency associated with low levels of NO, praliciguat decreased blood pressure levels and alleviated pathologies linked to NO deficiency across several target organs. At a level that produced little impact on overall BP, certain positive outcomes of praliciguat were noted. On the basis of these preclinical findings, the sGC stimulator praliciguat has the capability to offer numerous advantages for cardiovascular disorders with limited NO availability [29]. The effects of the activation of soluble guanylyl cyclase on vasodilation, inflammation, blood pressure regulation, and platelet aggregation are summarized in Figure 2.

In conclusion, the particular action mechanism of sGC stimulators may be advantageous, resulting in improved renal and cardiac function in HTN and preventing the evolution of related disorders [8].

### 2.3. Nonsteroidal Mineralocorticoid Receptor Antagonists (MRAs)

**Figure 3:**
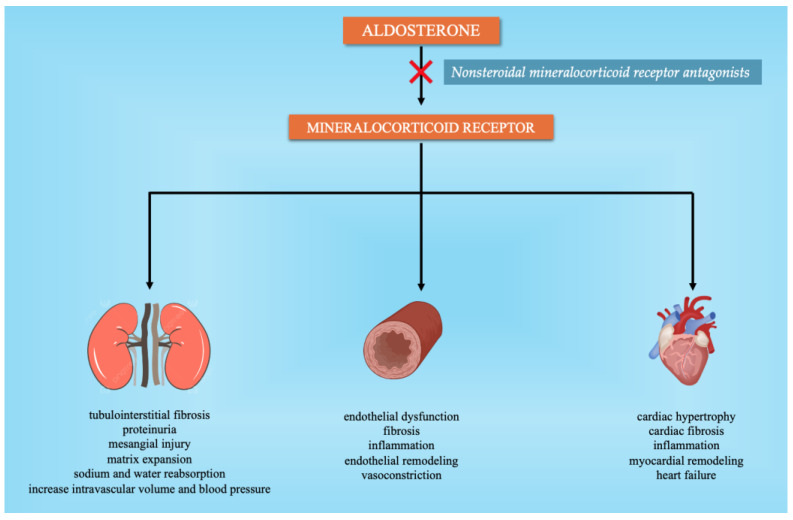
The role of nonsteroidal mineralocorticoid receptor antagonists in blood pressure control.

Mineralocorticoid receptor antagonists (MRAs) are valuable medications in guideline-based pharmacological treatment for cardiovascular disorders, including chronic heart failure with reduced ejection fraction, as summarized in Figure 3. Additionally, numerous clinical studies have demonstrated that steroidal MRAs such as spironolactone and eplerenone reduce BP in patients with RHNT [30,31,32]. The use of these medications is limited mainly because of the increased incidence of side effects like hyperkalemia [33]. As a result, alternative nonsteroidal MRAs are required to minimize the negative effects of steroidal antagonists [8]. Finerenone, esaxerenone, and apararenone are representatives of nonsteroidal dihydropyridine-based third- and fourth-generation MRAs, with two additional nonsteroidal MRAs, Balcinrenone (AZD9977) and Ocedurenone (KBP-5074), having recently entered clinical studies [34]. Finerone’s antihypertensive effectiveness has not been accurately assessed since no full dosage range studies have been carried out to determine its BP-reduction efficacy in hypertensive individuals [34], although a study on transgenic mice concluded that finerenone suppresses mineralocorticoid receptor-mediated fibrotic remodeling in cardiac fibroblasts [35]. The antihypertensive effect of esaxerenone, a nonsteroidal MRA authorized in Japan for the treatment of arterial HTN, was evaluated in many phase II clinical trials. In 426 Japanese patients with essential HTN treated for 12 weeks with various doses of esaxerenone (1.25 mg/2.5 mg/5 mg daily), SBP was reduced dose-dependently by −10.7, −14.3, and −20.6 mmHg, versus a decrease of −7.0 mmHg in the placebo group [36].

In addition, the safety and effectiveness of esaxerenone were evaluated in two multicenter, open-label, non-randomized dose-escalation trials in Japanese hypertensive patients with moderate renal dysfunction (eGFR ≥ 30 and <60 mL/min/1.73 m^2^). The main endpoints were modifications in SBP and DBP from baseline after 12 weeks of therapy, and, in both trials, esaxerenone as monotherapy or as an addition to RAAS inhibitor treatment showed significant antihypertensive benefits [37]. In the ESAX-HTN phase III clinical trial, 1001 patients with HTN were randomly assigned to receive either esaxerenone 2.5 or 5 mg daily or eplerenone 50 mg daily for 12 weeks. While the low dose of esaxerenone resulted in comparable decreases in BP as eplerenone, the higher dose was substantially more efficient at BP reduction. The plasma half-life of 18.6 ± 2.4 h may be one possible explanation for its enhanced antihypertensive effectiveness [38]. KBP-5074, a new and highly selective nonsteroidal MRA, dose-dependently decreased BP and 24-h urinary albumin excretion in the Dahl salt-sensitive hypertensive rat model [39]. In a multicenter, randomized, double-blind, placebo-controlled BLOCK-CKD trial, the safety and effectiveness of KBP-5074 were assessed in 162 patients with RHTN and advanced CKD (stage 3b/4). KBP-5074 substantially decreased SBP at the completion of the study, and the placebo-subtracted treatment difference was −7.0 mmHg with a KBP-5074 of 0.25 mg and −10.2 mmHg with the higher dose [40].

Novel treatment strategies, including nonsteroidal MRAs, may serve as a safer solution for HTN management in patients with hypertension-related complications without the need for a potassium-lowering drug. Unquestionably, research in this field might considerably help the continuing development of safe and efficient new MR antagonists [8].

### 2.4. Sodium/Glucose Cotransporter-2 Inhibitors (SGLT2i)

Sodium/glucose cotransporter-2 inhibitors (SGLT2i) are a novel class of glucose-lowering medications that inhibits the sodium-glucose cotransporter 2 found on the apical membrane of the proximal convoluted tubules [41], resulting in suppressed glucose reabsorption and increased urine glucose excretion [42]. Even though the main endpoint of the clinical trials involving SGLT2i was glucose regulation, the impact on BP and body weight were extensively described as secondary endpoints and safety outcomes [43]. Retrospective assessment of clinical trials has shown that SGLT2i has a positive, long-term effect on both SBP and DBP [44]. The postulated mechanisms involved in BP lowering are associated with the natriuretic effect [45], alterations of the renin–angiotensin–aldosterone system [46], improved arterial stiffness [47,48], reduced sympathetic system activity (which is overexpressed in response to hyperglycemia) [49], improved circadian rhythm [50], reduced oxidative stress, and potentially improved endothelial dysfunction [51,52]. A meta-analysis of 45 placebo-controlled trials found a mean drop in SBP of −3.77 mmHg, while six active-controlled studies found a mean drop in SBP of −4.45 mmHg and −2.01 mmHg for DBP in comparison to other drugs [53]. Another meta-analysis including 22,528 patients from 43 randomized control trials showed that SGLT2i reduced SBP by 2.46 mmHg (weighted mean difference) and DBP by 1.46 mmHg (weighted mean difference), which was significantly better than a placebo or other agents [54]. A meta-analysis of 20,980 patients from six studies that incorporated ambulatory BP monitoring showed a 24-h ambulatory SBP decrease of −3.76 mmHg and of −1.83 mmHg regarding the DBP, validating prior observations that the 24-h ambulatory BP is a stronger predictor of cardiovascular risk and mortality than office BP [55]. Empagliflozin appears to lower night-time BP to a smaller degree than daytime or 24-h SBP and DBP without a rise in heart rate (HR) and regardless of the administration of a diuretic or ACE/ARB [56]. In a placebo-controlled, double-blind study of the impact of canagliflozin on type 2 diabetes mellitus inadequately controlled with metformin monotherapy, a decrease in SBP was observed varying from −0.9 mmHg with 50 mg canagliflozin once daily to −4.9 mmHg with 300 mg canagliflozin once daily, in comparison to −1.3 mmHg with a placebo and −0.8 mmHg with sitagliptin [57]. In another placebo-controlled, double-blind, phase III study, patients with uncontrolled type 2 diabetes and HTN, under treatment with oral antihyperglycemic drugs, insulin, or both, as well as a ≥1 antihypertensive drug, were randomized (1:1) to dapagliflozin 10 mg once daily or to a placebo. Dapagliflozin 10 mg considerably lowered BP and HbA1c and was comparable to a placebo in terms of tolerability [58]. Patients who were previously taking a β-blocker or calcium-channel blocker benefited the most from its blood pressure-lowering effects. Dapagliflozin might be beneficial for patients with type 2 diabetes who need a diuretic-like action to improve BP management, hence enhancing the effectiveness of antihypertensive medication regimens [58].

SGLT2is are promising drugs that ameliorate cardiovascular and renal morbidity and mortality [59], and because of their impact on BP, they should be regarded as a second-line treatment for patients with diabetes or cardiovascular disease who also have HTN [44].

### 2.5. Aminopeptidase of the Brain Renin-Angiotensin System

There are now several classes of antihypertensive drugs available to manage BP, including RAAS blockers, beta-blockers, calcium-channel blockers, and diuretics, but despite the extensive availability of antihypertensive medications, a fraction of people persist in having poorly managed HTN [60,61]. The activation of RAS in the brain enhances sympathetic tone, resulting in an increase in vascular resistance and the release of arginine vasopressin, both of which contribute to a raised blood pressure level [62,63,64,65].

Angiotensin II (Ang II) and angiotensin III (Ang III) are the primary physiologically active compounds of the brain RAS [66]. Aminopeptidase A (APA), a membrane-bound zinc metalloprotease, is involved in Ang II’s N-terminal cleavage, converting Ang II into Ang III [67]. Intracerebroventricular Ang II and Ang III elevate BP via three potential mechanisms: (i) synaptic suppression of the baroreflex in the tractus solitarius nucleus, (ii) increased activity of the sympathetic nervous system, and (iii) the release of arginine-vasopressin (AVP) into the circulatory system, 4 [68]. An experimental study showed that Ang III produced by APA is one of the primary effector peptides of the brain RAS, exerting stimulatory tonic modulation over BP in conscious hypertensive rats [69]. Therefore, selective suppression of brain Ang-III production with an APA inhibitor is a feasible method for lowering BP in hypertensive patients [70]. However, orally or systemically administered EC33, a selective APA inhibitor, does not cross the blood–brain barrier [71,72]. Firibastat (formerly termed RB150) and NI956, the only two oral prodrugs of selective brain APA inhibitors EC33 and NI929, can penetrate the blood–brain barrier, where the disulfide bridges are broken off by brain reductases to generate the active molecules [73,74]. From there, the molecules EC33 and NI929 suppress the brain’s APA activity, prevent the production of brain Ang-III, diminish the release of AVP into circulation, and lower the mean arterial BP, without altering the HR [71,72,73,74,75,76]. Various preclinical research has revealed that brain APA inhibitor prodrugs are very safe and reliable at lowering BP in hypertensive animal models [71,72,73,75,77]. The antihypertensive impact of firibastat in experimental models of hypertension has been linked to three distinct mechanisms: (1) a reduction in vasopressin release from the posterior pituitary into the circulation, resulting in enhanced diuresis and lowered extracellular volume; (2) a reduction in sympathetic tone, and thus, lowering vascular resistance; and (3) an improvement in baroreflex activity [73,75,76,78,79]. Published phase I–II trials of firibastat showed its safety and clinical efficacy in lowering BP in hypertensive patients without affecting systemic RAS parameters or vital signs [80,81,82]. However, this finding should be regarded with care, due to limitations that include the modest number of phase II clinical trials (n = 2), the small sample size of patients, and the absence of a head-to-head assessment between firibastat and the standard-of-care antihypertensive medications [70]. A phase III clinical trial (FRESH) evaluated the safety and effectiveness of firibastat versus a placebo in 502 patients with uncontrolled or resistant primary HTN but failed to demonstrate efficacy in lowering unattended office systolic BP [83]. Firibastat was the first prodrug of EC33 with centrally acting APA inhibitor activity [72]. Recently, Keck et al. announced the discovery of NI956, a novel centrally active brain APA inhibitor prodrug of NI929 [74]. The authors demonstrated that NI929 was ten times more potent and effective than EC33 in inhibiting brain APA enzymatic activity in vitro and in vivo in hypertensive rats, but it has yet to undergo phase I–III clinical trials [74].

### 2.6. Vasoactive Intestinal Peptide (VIP) Receptor

Experimental research demonstrated that the vasoactive intestinal peptide (VIP), a neuropeptide that performs as a neurotransmitter and neuromodulator and impacts the function of various organ systems, is also responsible for reducing BP, increasing the heart rate and myocardial contraction, and decreasing vascular resistance [84,85].

**Figure 4:**
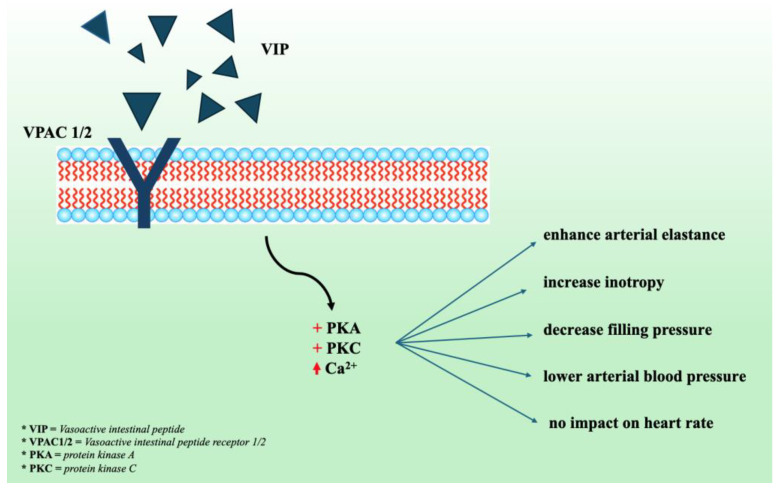
The effects of vasoactive intestinal peptide receptor activation on cardiovascular system; VIP—vasoactive intestinal peptide; VPAC 1/2—vasoactive intestinal peptide receptor type 1 and 2; PKA—protein kinase A; PKC—protein Kinase C; Ca^2+^—calcium.

VIP induces positive inotropic, chronotropic, and vasodilatory actions through the activation of two G protein-coupled receptors VPAC1 and VPAC2 [86]. VIP is an important therapeutic target for systemic and pulmonary hypertension, as well as heart failure, because of its connection with various cardiovascular and cardiopulmonary illnesses [84]. Vasomera was produced by combining a VIP analog with a polypeptide with elastin-like properties. This medication binds selectively to VPAC2, resulting in limited intestinal adverse effects caused by VPAC1 activation, which are summarized in Figure 4. Vasomera has a longer half-life than native VIP, making it a superior therapeutic agent [87]. A study involving bilateral nephrectomy-induced hypertension in rodent models revealed that Vasomera enhanced arterial elastance and inotropy and decreased filling pressures [88]. Two phase I randomized, double-blind, placebo-controlled trials investigating the safety, tolerability, pharmacokinetics, and hemodynamic response of this drug in patients with essential hypertension. The authors concluded that Vasomera dose-dependently lowers both SBP and DBP, with no clinically relevant dose-dependent changes in HR [89], though the final results have not yet been published.

### 2.7. Intestinal Sodium (Na^+^)/Hydrogen (H^+^) Exchanger 3 (NHE3)

**Figure 5:**
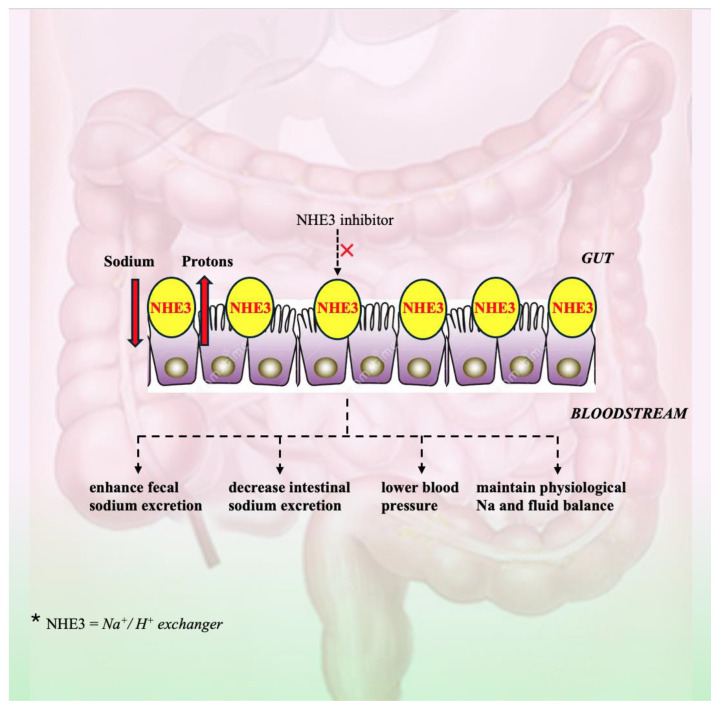
The effects of Na/H exchanger inhibition on blood pressure control; NHE3—Na^+^/H^+^ exchanger; 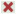
—inhibition of.

Effective salt limitation is a suggested lifestyle modification as part of hypertension treatment, although it is difficult to implement on an individual basis, with many patients being unable to adopt major and significant dietary changes [90]. Extensive renal studies indicated that patients’ salt intake substantially exceeded the recommended 5–6 g per day [91]. Recently, non-absorbable intestinal sodium (Na^+^)/hydrogen (H^+^) exchanger 3 (NHE3) inhibitors emerged as a promising target in this field. These inhibitors are designed to lower intestinal sodium absorption and facilitate the otherwise difficult lifestyle modification of adopting a low-sodium diet, as summarized in Figure 5 [90].

NHE3 inhibitors have specific effects on hypertensive and heart failure patients and, particularly, in patients with heart failure where the diuretic treatment becomes progressively ineffective or patients develop diuretic resistance and/or concurrent renal failure and hypokalaemia [92]. NHE3 is one of the most significant Na^+^/H^+^ antiporters in the small intestines and proximal renal tubules [93]. Multiple lines of evidence emphasize the capability of NHE3 in preserving physiological Na^+^ and fluid balance, basal BP homeostasis, and the pressure–natriuresis response [93]. Lowering GI sodium uptake by inhibiting NHE3 may be an efficient approach for managing HTN [94,95]. NHE3 may influence the natriuretic effects of empagliflozin, a proximal tubule SGLT2 inhibitor that decreases BP [96]. In an experimental study, the NHE3 inhibitor SAR218034 (SAR) enhanced fecal sodium excretion, decreased urine sodium excretion and intestinal sodium uptake, and significantly decreased SBP in rats [94]. Furthermore, when SAR is administered with the ACE inhibitor ramipril, its hypotensive action is significantly improved. SAR had a similar antihypertensive impact on hypertensive, obese, and hyperinsulinemic rats [87]. Tenapanor, a new targeted NHE3 inhibitor, has been found to lower BP, fluid volume, albuminuria, and left ventricular hypertrophy in rats with nephrectomized kidneys and high salt intake [97]. Additionally, combining enalapril and tenapanor decreased cardiac diastolic dysfunction and arterial pulse wave velocity in comparison to enalapril in monotherapy [97]. Both medications, which are nonabsorbable after oral administration and solely target NHE3 in intestinal apical membranes, demonstrated barely modified plasma concentrations, suggesting limited systemic effects and enabling other NHE proteins to function normally [94,97]. By selectively blocking NHE3 and Na^+^ uptake from the gastrointestinal system, NHE3 inhibitors may be appealing for the treatment of RHTN in elderly individuals with constipation when paired with current antihypertensive medications. Nevertheless, tenapanor is not recommended in young individuals due mainly to salt loss and decline in BP [97]. With a different mechanism of action, AVE-0657 passes from the digestive tract into circulation after oral administration and is filtered by the glomerulus, where it suppresses the apical membrane NHE3 in the kidney, mainly the proximal tubules and much less the ascending limb of the loop of Henle [98,99,100]. Recent evidence demonstrated that AVE-0657 did not enhance gastrointestinal Na^+^ excretion, indicating that it does not suppress NHE3 in the small intestines like SAR218034 and tenapanor. Furthermore, AVE-0657 caused natriuresis and substantially lower blood pressure in Ang II-infused, high-salt-fed rats. When the ARB losartan was administered in combination with the AVE-0657 in Ang II and high salt intake induced hypertension, the blood pressure was restored to control levels [99,100].

### 2.8. Endothelin Receptor (ETR)

**Figure 6:**
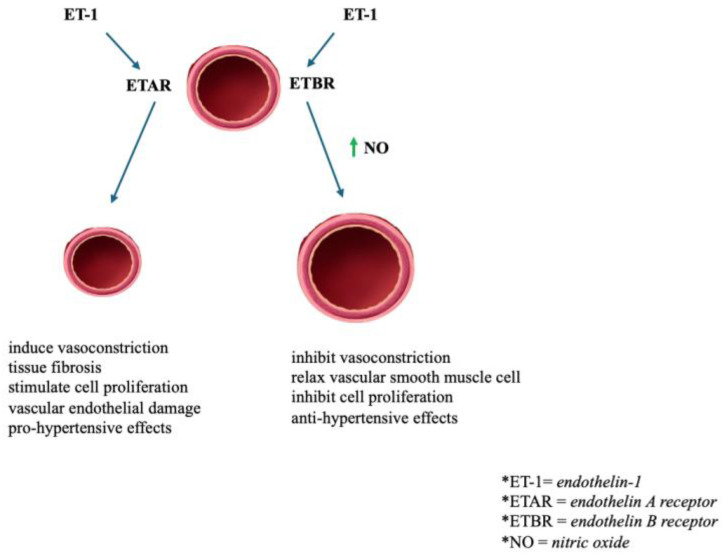
The effects of endothelin receptor A and B activation on vasculature; ET-1—endothelin-1; ETAR—endothelin A receptor; ETBR—endothelin B receptor; NO—nitric oxide.

Endothelin-1 (ET-1), an endothelium-derived contractile agent secreted by vascular endothelial cells, is a powerful vasoconstrictor peptide and a key factor in regulating vascular tension [101]. Taking into account the possible benefits of suppressing the endothelin system in cardiovascular illnesses such as heart failure and pulmonary hypertension, the discovery of endothelin receptors (ETAR and ETB) on the basis of their molecular structures as well as their pharmacological responses to different agonists and antagonists has been a significant milestone [102]. These two receptors are broadly dispersed and expressed in many tissues, with a ratio of organ-dependent ETA/ETB receptors, ETB receptors being more expressed in the brain and kidneys, and ETA receptors being featured prominently in all blood vessels [103]. This characteristic may be decisive for the interpretation of organ-specific feedback to the endothelin receptor antagonist (ETRA). Endothelin receptors in the cardiovascular system have a highly diverse and opposite effect. Stimulation of the ETA receptor (ETAR) generates a vasoconstrictor effect in both large arteries and small resistance vessels, leading to the maintenance of the basal vascular tone [104]. In contrast, stimulation of the vascular ETBR results in a vasodilator effect owing to the production of endothelial factors including NO and prostacyclin [105]. Therefore, ETBR appear to counteract the vascular response of ETAR. However, in pathological processes, this divergent result may be modified, hence why, in certain conditions, a non-selective blockade of both ETAR and ETBR may be preferable to a selective ETAR blockade [106]. The coupling of ET-1 to ETAR may induce vasoconstriction, cell proliferation, tissue fibrosis, and vascular endothelial damage, all of which contribute to the development of HTN summarized in Figure 6 [107]. The coupling of ET-1 to ETBR may stimulate endothelial cells to generate NO, which relaxes vascular smooth muscle and inhibits vasoconstriction and cell proliferation [108]. Consequently, suppression of ETAR might be a therapy option for arterial HTN. Currently, several ETRAs have been described, and they are classified into three categories: selective ETAR antagonists including darusentan and ambrisentan; nonselective ETARs including bosentan and macitentan; and selective ETBR antagonists [104].

The antihypertensive effect of bosentan, the first ETRA studied in clinical trials, at dosages of 0.5 or 2.0 g/day in patients with HTN was equivalent to that of enalapril, according to various studies [109]. A multicenter, randomized, double-blind, parallel-group dose-response study showed that darusentan, a selective ETAR antagonist, dose-dependently decreased BP in patients with HTN [110]. A meta-analysis of 18 studies including 4898 patients investigating the efficacy and safety of ETRAs revealed that they substantially decreased 24-h ambulatory BP, but they were associated with more side effects than a placebo [111]. Two large trials, DORADO [112] and DORADO-AC [113], were conducted on darusentan for the treatment of RHTN. In the first trial, the addition of darusentan led to a considerable reduction in BP in patients with RHTN, demonstrating the effectiveness of endothelin receptor suppression in this clinical setting [114]. Nonetheless, in the second trial, the placebo and darusentan did not vary substantially after 14 weeks regarding the main endpoints, especially sitting office BP. Darusentan was considerably superior to a placebo and guanfacin in decreasing BP in these refractory patients, as shown by 24-h ambulatory monitoring of BP. Consequently, since the predetermined co-primary endpoints were not met, the trial was discontinued [115]. Aprocitentan, the active metabolite of macitentan, is the subject of the only continuing hypertension research designed to demonstrate a therapeutic benefit for patients with RHTN. Aprocitentan is a powerful, orally active, dual ET receptor antagonist that inhibits the binding of ET-1 to both ETA/ETB receptors at a ratio of 1:16 [116,117,118]. Aprocitentan has proven to have a synergistic effect with RAAS blockers, causing a dose-dependent reduction in BP. Therefore, it provides a promising novel therapy option for instances in which high blood pressure cannot be successfully managed with existing therapies [119]. Thus far, three hypertension-related clinical trials have been conducted. One study aimed to determine the blood pressure-lowering potential of aprocitentan when administered in addition to antihypertensive treatment in patients with uncontrolled BP and chronic renal disease. Surprisingly, it was decided for non-medical reasons not to begin this research [120]. The purpose of another study, a double-blind, parallel trial, was to assess the dose response of aprocitentan (four doses administered once daily) on SBP and DBP in patients with grade 1 or 2 essential hypertension. Aprocitentan at 10, 25, and 50 mg substantially lowered sitting office SBP/DBP from baseline to week 8, in comparison to lisinopril at 20 mg, which decreased BP by 4.84/3.81 mmHg. The maximal result was obtained at a dosage of approximately 30 mg, whereas half of the impact was achieved at a dose of approximately 10 mg, with the dose-response plateauing between 25 and 50 mg. In addition, aprocitentan was not related to neurohumoral system activation. The incidence of major adverse outcomes was comparable across the aprocitentan and placebo groups [121]. The objective of the PRECISION trial was to assess the BP-lowering efficacy of aprocitentan when combined with other antihypertensive medications in patients with RHTN [122]. The results showed that aprocitentan was well tolerated and superior to a placebo in decreasing blood pressure at week 4, with a maintained effect at week 40 in individuals with resistant hypertension [123].

### 2.9. Dual L-Type Calcium Channel Blocker/Endothelin A/B2 Receptor Antagonist

It is well established that an increased vascular tone mediated by the sympathetic nervous system (SNS) and the renin–angiotensin pathway significantly contribute to the physiopathology of HTN [124]. Vascular tone is mostly reliant on the intracellular Ca^2+^ levels in vascular smooth muscle cells, which are modulated by the endothelium, a significant regulator of vascular reactivity and BP [125]. The endothelium secretes vasodilators, such as prostaglandin I2 (PGI2), endothelium-derived hyperpolarizing factor (EDHF), and NO, along with vasoconstrictors, such as thromboxane A2, Ang II, and endothelin (ET) [126,127,128]. Vascular tone is regulated during physiological conditions by the balance between vasodilator and vasoconstrictor signals [129]. In pathophysiological states, however, a reduction in endothelium-derived vasodilators and a rise in endothelium-derived or adipose- and inflammatory-tissue-derived vasoconstrictors enhance vascular tone and BP, resulting in HTN [101]. Even though it is assumed that various factors lead to essential hypertension, the main objective is always enhanced vascular tone, which is regulated by elevated Ca^2+^ input through L-type Ca^2+^ channels in vascular smooth muscles [130]. Targeting the L-type calcium channel using calcium channel blockers successfully decreases BP in hypertensive patients [131]. ET-1 is an important focus for unfulfilled medical requirements in the management of uncontrolled hypertension by standard therapy [125]. Upon activation of its type A and type B2 receptors, ET-1 is a strong vasoconstrictor and inflammatory mediator. On the other hand, the B1 receptor exerts vasodilatory and anti-inflammatory properties [131]. It is well established that prolonged rises in BP are related to cumulative endothelial dysfunction, resulting in an increased ET-1 release in cardiovascular tissues in patients with HTN [125,132]. Consequently, the suppression of ET-1 synthesis and ETA/B2 receptor-mediated activity may represent a significant method for reducing BP and preventing vascular and cardiac damage [133]. Recent research discovered that sargachromenol-D, a dual L-type calcium channel blocker/ET A/B2 antagonist, obtained from the marine brown alga *Sargassum siliquastrum*, lowers ET-1 and K^+^ depolarization-induced vasoconstriction in rabbit basilar arteries and lowers BP in rodent models of hypertension. More research is required to determine the possible significance of sargachromenol-D in the management of hypertension in humans [131].

### 2.10. Drugs Targeting the NO Pathway

NO, a molecule primarily produced by the vascular endothelium, stimulates guanylyl cyclase to synthesize 3,5-cyclic guanosine monophosphate, which leads to vasodilation of vascular smooth muscle cells; prevents platelet adhesion and aggregation; and has anti-inflammatory, antiproliferative, and anti-migratory implications on leukocytes, endothelial cells, and vascular smooth muscle cells, thereby protecting against atherosclerosis [134]. In HTN, the fundamental feature of endothelial dysfunction is reduced NO bioavailability [135,136,137]. Hypertensive individuals have more reactive oxygen species (ROS), which deplete NO and reduce its bioavailability [134]. Shear stress and other elements trigger the vascular endothelium to produce and release NO into adjacent tissues and cells, lowering BP by decreasing vascular tone and smooth muscle cell proliferation. These findings imply that raised NO concentrations in the body may lower BP [138]. The bioactive lipid mediator sphingosine-1-phosphate is a powerful stimulator of endothelial NO synthase via G protein-coupled receptors [139]. The autocrine/paracrine activation of the sphingosine-1-phosphate receptor (S1PR) is critical to the control of BP, S1PR1 signaling being a crucial mechanism in BP homeostasis. In an experimental study, the administration of NOS substrates like L-arginine or its precursor L-citrulline reduced BP in hypertensive rats [140]. Moreover, in a clinical study, the administration of 12 g of L-arginine daily for four weeks in hypertensive patients substantially lowered both SBP and DBP [141]. Drugs that lower ADMA and SDMA concentrations, including resveratrol, melatonin, and N-acetylcysteine (NAC), may also help regulate BP [142,143,144]. Additional medications related to the NO pathway might be identified and implemented to treat HTN in the near future.

### 2.11. Dopamine β-Hydroxylase (DβH) Inhibitor

Dopamine β-hydroxylase (DβH), an essential component in the catecholamine biosynthesis pathway, which catalyzes the hydroxylation of dopamine to produce noradrenaline in the SNS, is a candidate for the management of HTN as well as other sympathetically activated cardiovascular diseases, including HF [145]. Suppression of DβH has theoretical benefits over the adrenergic receptor blockade: (a) it induces progressive rather than abrupt sympathetic inhibition and (b) it improves dopamine availability, resulting in renal vasodilation, natriuresis, and diuresis [146]. On the premise of the importance of DβH in managing HTN, four major categories of DβH inhibitors have been identified and tested to date: (1) copper chelator-based inhibitors: disulfiram and fusaric acid [147,148]; (2) thiocarbonyl encompassing heterocyclics as inhibitors: imidazolidine, tetrazole, and benzimidazole derivatives as the top alternative; (3) mechanism-based inhibitors: 2-thiophen-2-ylamine was the most effective inhibitor from this class; and (4) imidazole 2-thione-based inhibitors: nepicastat, etamicastat, and zamicastat [149]. Despite the considerable number of DβH inhibitors discovered, only a few drugs decrease BP in humans [150]. First, second, and early third-generation DβH inhibitors, including disulfiram, fusaric acid, and nepicastat, mainly lacked efficacy or selectivity for DβH or induced significant central nervous system-related side effects, rendering them therapeutically ineffective [151]. Etamicastat (BIA 5–453) is a powerful and reversible DβH inhibitor that cannot cross the blood–brain barrier and is hence selective for peripheral DβH when orally administrated [146]. Etamicastat reduces BP in SHR rats but not in normotensive WKY (Wistar Kyoto) rats and lengthens survival in HF animal models [152]. In studies involving healthy males and individuals with mild to moderate HTN, 24-h ambulatory BP was reduced dose-dependently [146]. A double-blind, randomized, placebo-controlled clinical trial of zamicastat, with mixed single and multiple escalating dosages, assessed the safety, tolerability, and pharmacokinetic and pharmacodynamic characteristics of the drug [153]. Over the last few years, etamicastat and zamicastat have helped DβH achieve attention as a potential antihypertensive therapeutic target. Animal models and human clinical trials also show that DβH is a legitimate target for the treatment of HTN, requiring the development of novel inhibitors to address the limitations [150].

### 2.12. Ouabain Inhibitors

Throughout the last two decades, research extending from the entire body to the gene level has led to a better understanding of the clinical significance of two pathways [154] that modulate the renal Na^+^/K^+^ pump activity through endogenous ouabain (EO), a salt-modulating hormone whose circulating concentrations are genetically regulated [155,156,157], and through the polymorphism of the genes responsible for the cytoskeletal protein adducin [158,159,160]. Ouabain attaches and stimulates Na^+^/K^+^ ATPase, triggering a signaling cascade that inhibits Na^+^ and K^+^ flux and activation of cytoplasmic tyrosine kinase (cSRC), leading to inflammation and ROS accumulation in the vasculature [161]. cSRC initiation may lead to HTN and HF, and ouabain has been found to enhance vascular resistance, resulting in HTN in rats [162]. Therefore, ouabain inhibitors have been explored as a possible future for the treatment of arterial HTN. Rostafuroxin, which was designed to inhibit the effect of ouabain on Na^+^/K^+^ ATPase, has been demonstrated to decrease SBP, enhance endothelium-mediated arterial relaxation, augment NO synthesis, and decrease oxidative stress in the resistance arteries of DOCA salt-hypertensive rats [163]. The PEARL-HT clinical trial, a phase 2b multicenter, double-blind, four arms, parallel-group, and active comparator-controlled study, performed in recently identified yet never treated (naive) hypertensive patients, compared rostafuroxin to the generic drug losartan. The major conclusion of this clinical trial was that patients with the genetic profile P2a or LSS AA genotype responded more favorably (greater SBP drop) to rostafuroxin 50 μg than to losartan [164]. Additional research is required to determine the applicability of rostafuroxin as a possible therapy for HTN in humans.

### 2.13. Leptin

Obesity is associated with a variety of unfavorable metabolic and cardiovascular consequences, being a leading factor of primary hypertension. To date, the pathophysiology of obesity-related HTN has not been completely understood [165]. The increasing prevalence of cardiovascular events in obese individuals has prompted researchers to determine the neurohormonal impact of leptin on the cardiovascular system [166]. A positive correlation between the amount of circulating leptin and HTN and associated cardiovascular morbidity was demonstrated [167,168,169]. Kramer et al. identified elevated leptin concentrations in elderly hypertensive patients and concluded that an increased leptin concentration raises the likelihood of developing HTN by 70% [170]. According to extensive studies, leptin is a key link connecting fat, sympathetic overdrive, and HTN. Leptin is a 16 kDa neuroendocrine hormone that regulates food intake, metabolic activity, and fat development. In addition, leptin promotes sympathetic nerve activity in a variety of tissues, including those implicated in cardiovascular homeostasis, kidneys, and blood vessels [171], and it has been demonstrated that these sympathoexcitatory actions of leptin elevate arterial pressure [172]. Furthermore, transgenic mice that overexpress leptin irrespective of adipose tissue mass exhibit sympathetically mediated HTN, indicating that there is a strong correlation between adiposity, sympathetic activity, BP increase, and leptin [173]. Leptin and HTN have a reciprocal connection. Leptin enhances arterial BP by stimulating aldosterone synthase (CYP11B2), thereby increasing aldosterone production from the zona glomerulosa. In contrast, hypertension inhibits the leptin-induced heart contractile response [174]. Exogenous administration of leptin is unable to properly manage energy homeostasis in the setting of obesity while preserving its impact on cardiovascular sympathetic output and BP. This “selectivity” in leptin resistance may elucidate how leptin induces HTN and sympathetic hyperactivity in obese individuals [175], which is further corroborated by the sympathoinhibition and hypotension caused in obese rabbits by central blockage of leptin signaling [176]. The removal of leptin receptors in the subfornical organ led to a reduction in BP [177]. Uncertainty persists regarding whether selective leptin antagonists possess antihypertensive properties, and further experimental studies are required.

### 2.14. Insulin-Resistant Aminopeptidase (IRAP)

A further potential target for the treatment of HTN is represented by insulin-resistant aminopeptidase (IRAP), the Ang IV receptor, which was discovered in 2001 [178]. Ang IV functions as a vasoconstrictor via the AT4 receptor in the basolateral artery of rats [179] and increases BP and decreases blood flow through the AT1 receptor [180]. As a result, Ang IV, its peptide analogs, and nonpeptide IRAP inhibitors have been evaluated in HTN experimental studies [8]. HFI-419, an IRAP inhibitor, represents a promising therapy for well-known cardiac disorders, resulting in improved target organ function [181]. HFI-419 is superior to candesartan cilexetil in antifibrotic effectiveness and renoprotection and to the ACE inhibitor, perindopril, in mouse kidney injury produced by high salt concentrations [182]. In addition, there is evidence that IRAP deletion in mice reduces lipid formation in the plaque region and prevents plaque rupture. These findings imply a close connection between angiotensin signaling and atherogenesis [183]. In future research, IRAP inhibitors may be effective for preventing cardiac and renal damage. To demonstrate the efficacy of IRAP inhibitors as a potential therapeutic approach for HTN management, more research is necessary [8]. All of this novel targets are also presented in Table 1.

### 2.15. Gastrointestinal Microbiota

The human gut contains a significant number of bacteria with a crucial role in human health [184,185]. Dysbiosis of the gastrointestinal microbiota is linked to HTN development, and gastrointestinal microbiota play a significant part in BP management [186]. Various human cross-sectional research has shown a link between gut microbiota and HTN [187,188,189,190,191,192,193,194,195,196]. In a study conducted in China on a cohort of 41 healthy subjects, 56 with borderline HTN, and 99 hypertensive patients, microbial density and variety were significantly lower in the pre-hypertension and hypertension groups when compared to the control group. Furthermore, microorganisms associated with health status were decreased, while substantial quantities of bacteria, including *Prevotella* and *Klebsiella* spp., were found [189]. As previously discovered, data from the cross-sectional HELIUS cohort research showed that *Klebsiella* spp. and *Streptococcaceae* spp. were positively linked with BP [192,194]. The fermentation of indigestible dietary fiber in the large intestine is one of the important roles of the human intestinal microbiota. This fermentation process produces short-chain fatty acids (SCFAs) such as acetate, propionate, and butyrate [197]. SCFAs primarily control BP by stimulating G protein-coupled receptors Olfr78 and Gpr41 in vascular or renal tissues. Olfr78 is localized in the renal juxtaglomerular apparatus and regulates renin production in response to SCFAs [198]. There have also been studies that show that an immediate SCFA bolus lowers BP in anesthetized rats and that this action is predominantly mediated through Gpr41 [199]. As a result, Olfr78 is now thought to be predominantly accountable for increasing BP, while Gpr41 is thought to be mostly accountable for reducing it [87]. A randomized, placebo-controlled trial found that *Lactobacillus helveticus* LBK-16H- fermented milk with bioactive peptides lowers BP in hypertensive subjects [200]. All of the findings suggest that lactobacilli may prevent the onset of HTN, and probiotic supplementation might be a possibly useful approach to treating HTN by reinstating the gut microbiota [87]. Further research into the mechanism of interaction between the gut bacteria and the host will be required in the future. To comprehend the therapeutic impact of probiotics on HTN, the effects of various probiotics, as well as the probable molecular processes responsible for improved BP, must be assessed [87].

### 2.16. Vaccines

Vaccines addressing the RAS for the management of HTN have been documented since the 1950s in the lengthy history of immunotherapy [201]. Ang I and Ang II vaccines, as well as Ang receptor-targeting vaccines, may effectively decrease BP in animal models. Furthermore, several clinical trials have been carried out, and although no substantial antihypertensive effects were observed, in both phase I and phase II clinical trials, the Ang I vaccine PMD3117 considerably elevated anti-Ang I antibody titers [202,203,204,205,206]. Thus, more research will be necessary to determine if higher titers may reduce BP [87]. In a multicenter, double-blind, randomized, placebo-controlled clinical study of the Ang II vaccine AngQb-Cyt006, 72 subjects with mild to moderate HTN underwent vaccination or were administered a placebo. In comparison to baseline values, SBP and DBP were lowered by −9.0 and −4.0 mmHg, respectively, in the 300 μg vaccination dosage cohort [207]. This research proved for the first time that a vaccine may have a hypotensive impact on humans, but this effect must be confirmed in a larger hypertensive population in the future [87].

**Table 1:** New targets in hypertension.

Target	Drug	Mechanism	Main Findings	Clinical Trial/Study
**NP, pGC-A**	CRRL269 MANP (ZD100) ANX042	pGC-A activator	- Vasorelaxation and antihypertensive properties in a canine model of ischemia-induced acute renal dysfunction - Effective at lowering BP, with considerable renal protective function and decreased aldosterone levels - Significant diuretic and natriuretic effects lacking vasodilatory hypotensive features - Higher decreases in office SBP and DBP and 24-h ambulatory SBP than valsartan alone	Chen et al. MANP-HTN-MS (completed 2020) Chen et al.
**Neprilysin+ Angiotensin II Receptor Blockers**	Valsartan/sacubitril (LCZ 696)	Angiotensin receptor-neprilysin inhibitors		NCT00549770 (completed 2015)
**Guanylyl cyclase A**	Praliciguat (IW1973)	Stimulator of guanylate cyclase	- Attenuated hypertension and NO deficiency-related diseases	Shea et al.
**Nonsteroidal mineralocorticoid receptors (MRAs)**	Finerenone Esaxerenone KBP-5074	Mineralocorticoid receptor antagonist	- Suppresses mineralocorticoid receptor-mediated fibrotic remodeling in mice cardiac fibroblasts - As monotherapy or as an addition to RAAS inhibitor treatment showed significant antihypertensive benefits - Dose-dependently decreased BP and 24-h urinary albumin excretion in the Dahl salt-sensitive hypertensive rat model - Substantially decreased SBP at the completion of the human study	Lavall et al. NCT02722265 (completed 2019) Chow et al. BLOCK-CKD (Phase 2b—completed 2024)
**Sodium/glucose cotransporter-2**	Canagliflozin Dapagliflozin Empagliflozin	SGLT2 inhibitor	- A decrease in SBP was observed. - Considerably lowered BP and HbA1c and was comparable to placebo in terms of tolerability - Reduced SBP and DBP versus placebo	NCT00642278 (completed 2013) NCT01195662 (completed 2016) EMPA-REG BP (completed 2016)
**Aminopeptidase A**	Firibastat (RB150) NI956	APA inhibitor	- The following was observed in experimental models of hypertension: (1) a reduction in vasopressin release from the posterior pituitary into the circulation, resulting in enhanced diuresis and lowered extracellular volume; (2) a reduction in sympathetic tone, and, thus, lower vascular resistance; and (3) an improvement in baroreflex activity. - In human trials, it failed to demonstrate efficacy in lowering unattended office systolic BP. - Ten times more potent and effective than firibastat in inhibiting brain APA enzymatic activity in vitro and in vivo in hypertensive rats	FRESH (completed 2022) Keck et al.
**Vasoactive intestinal peptide**	Vasomera (PB1046)	VIP inhibitor agonists	- Dose-dependently lowers both SBP and DBP, with no clinically relevant dose-dependent changes in HR	NCT01523067 (completed 2013)
**Na^+^/H^+^ exchanger 3 (NHE3)**	Tenapanor SAR 218034 AVE-0657	NHE3 inhibitor	- Lowers BP, fluid volume, albuminuria, and left ventricular hypertrophy in rats with nephrectomized kidneys that have been given a salty diet - Enhances fecal sodium excretion, decreases urine sodium excretion and intestinal sodium uptake, and significantly decreases SBP in rats - Causes natriuresis and substantially lowers hypertension in Ang II-infused, high-salt-fed rats	Spencer et al. Gao et al. Li et al./Zhuo et al.
**Endothelin receptor**	Bosentan Darusentan Aprocitentan	Nonselective ETR antagonists Selective ETR antagonist Dual ETAR/ETBR antagonist	- The antihypertensive effect of bosentan was equivalent to that of enalapril. - The addition of darusentan led to a considerable reduction in BP in patients with RHTN. - Placebo and darusentan did not vary substantially after 14 weeks regarding the main endpoints, especially sitting office BP. - It was well tolerated and superior to a placebo in decreasing blood pressure at week 4, with a maintained effect at week 40 in individuals with RHTN.	Krum et al. DORADO (NCT00330369-completed 2014) DORADO-AC (NCT00389779-completed 2014) PRECISION (NCT03541174- completed 2023)
**Dual L-Type Calcium Channel /Endothelin A/B2 Receptor**	Sargachromenol-D	Dual L-type calcium channel blocker/ET A/B2 antagonist	- Lowers ET-1 and K^+^ depolarization-induced vasoconstriction in rabbit basilar arteries and lowers BP in rodent models of hypertension	Park et al.
**Nitric oxide (NO)**	Sphingosine-1 phosphate (FTY702) L-arginine or L-citrulline	S1PR1 antagonist NO synthase activator	- Might enhance BP and worsen HTN in an ANG II rat model - May reduce BP in hypertensive rats	Gao et al. Dumont et al.
**Dopamine** **β** **-hydroxylase (D** **β** **H)**	Etamicastat (BIA 5–453)	Dopamine β-hydroxylase inhibitor	- In healthy males and individuals with mild to moderate HTN, 24-h ambulatory BP was reduced dose-dependently.	Almeida et al.
**Ouabain**	Rostafuroxin	Ouabain inhibitors	- Patients with the genetic profile P2a or LSS AA genotype responded more favorably (greater SBP drop) to rostafuroxin 50 μg than to losartan.	PEARL-HT
**Insulin-resistant aminopeptidase**	HFI-419	Insulin-resistant aminopeptidase (IRAP) inhibitor	- Superiority to candesartan cilexetil in antifibrotic effectiveness and renoprotection and to the ACE inhibitor, perindopril, in mouse kidney injury produced by abnormally high salt concentrations	Gaspari et al.

## 3. Other Possible Targets in Hypertension

### 3.1. Chemerin

Chemerin is known as a chemokine as a result of its first discovery within the immune system, where it stimulates plasmacytoid dendritic cells, natural killer cells, and tissue macrophages [208]. In 2007, chemerin was found to be an adipokine [209,210,211], with the primary synthesis in the liver and adipose tissue, which has favorable correlations with blood pressure, blood glucose, and obesity [212]. Chemerin is contractile in the vasculature of both humans and rats, with effects on the endothelium [213,214], smooth muscle cells [215,216], and local nerves [217], according to scientific literature. Chemerin enhances ROS in endothelial cells, it may reduce NO release [214,218,219], it is a mitogen in vascular smooth muscle cells and raises BP [220], and it also induces angiogenesis in the microvasculature [221,222]. The contraction of isolated arteries and the action of proven vasoconstrictors such as ET-1 mediate the impact of chemerin on BP levels [217,218,223]. Considering the vasoactivity of chemerin in vitro, little research has investigated its involvement in BP control. One study demonstrated that antisense oligonucleotide (ASO)-mediated decreases in whole-body chemerin in rats were associated with modest but statically relevant reductions in BP [224]. Another research showed that while hepatic chemerin predominates in circulating plasma, it does not contribute to blood pressure regulation. The blood-pressure-regulating chemerin protein must be derived from another source, and adipose tissue may be the one [208]. Nevertheless, specialized medications targeting chemerin require additional research.

### 3.2. Autophagy

Autophagy is an evolutionarily conserved catabolic process that preserves cellular homeostasis under many circumstances [225,226]. Autophagy is essential under normal settings to degrade long-lived proteins and defective organelles. Under situations such as hunger and hypoxia, autophagy is triggered, improving cell viability by generating energy sources via the decomposition of cellular components and by removing malfunctioning organelles [227]. Nevertheless, prolonged and unrestrained autophagic activation may result in the degradation of crucial molecules and organelles, which induces autophagic cell death [228,229]. Autophagy is involved in various pathogenic processes associated with hypertension and organ damage [230]. Regardless of significant advances in the knowledge of autophagy’s molecular mechanism, the comprehension of its adaptive versus maladaptive effects faces significant challenges [231]. A thorough comprehension of the pathogenic mechanism of autophagy that modulates HTN, particularly the organ damage induced by hypertension, could provide novel insights and strategies for HTN prevention and management. Inhibiting autophagy is not seen as a beneficial approach to managing hypertension. Conversely, preserving or augmenting autophagic action seems to promote cardiovascular health and may assist in the regulation of hypertension. It is crucial to acknowledge that the link between autophagy and hypertension is complex, and treatment strategies should focus on restoring or preserving autophagic equilibrium rather than merely blocking it [230].

### 3.3. Acetylation

Acetylation, as a component part of a class of post-translational modification (PTM) processes, modulates gene expression and transcription [232]. Hypertension requires the acetylation and deacetylation of functional proteins. By increasing histone 3 acetylation on the promoters of mineralocorticoid receptor target genes, suppression of histone deacetylases might decrease cardiac hypertrophy and fibrosis in spontaneously hypertensive rats [233]. Inhibition of lysine deacetylases also enhanced mineralocorticoid receptor acetylation and improved BP [234]. Hyperacetylation of mitochondrial SOD2 and increased production of oxidative stress contribute to endothelial dysfunction, vascular inflammation, and high blood pressure in mice when SIRT3 is depleted [235]. Histone acetyl-transferases (HATs) and histone deacetylases (HDACs) are key enzymes primarily involved in the modulation of lysine acetylation concentrations, hence proposing potential pharmacological targets for the treatment of cardiovascular disorders [232].

### 3.4. Angiogenesis

HTN can arise from functional and structural disturbances in the microvascular network, partially due to aberrant modulation of vascular endothelial growth factor (VEGF), a prominent angiogenic agent. Emerging results from research studies on anti-VEGF drugs indicate that VEGF serves not only as a trigger for proliferation and migration but also as a maintenance and protective agent for endothelial cells, with its dysregulated expression potentially disrupting vascular homeostasis. In hypertensive individuals, increased VEGF levels were found to link with cardiovascular risk, early microvascular impairment, and target organ dysfunction [236]. Research has postulated that Cluster of Differentiation (CD) 93 may be involved in the regulation of systemic and pulmonary hypertension. Additional confirmation of these data and theories could potentially help in identifying key components of the prospective targets for future treatment approaches [237].

### 3.5. Antioxidative Nutraceuticals Supplementation

Recognized nutritional and behavioral-associated risks for HTN, including excessive sodium consumption, excessive drinking habits, and a lack of physical activity, substantially lead to the widespread presence of this disorder. Supplementary nutritional imbalances are frequently associated with the onset of HTN, notably inadequate eating of fruits and vegetables, insufficient lactose consumption, and poor diets containing fatty fish. Recently, emphasis has been given to deficits of particular micronutrients, including folate, riboflavin, vitamin C, and vitamin D, as determinants of hypertension [238,239]. In addition to the established impacts on BP of the Dietary Approaches to Stop Hypertension (DASH) [240] and the Mediterranean diet [241], a variety of research focused on the potential BP-lowering effects of various nutritional changes within the supplements and nutraceuticals, predominantly including antioxidant compounds characterized by good tolerance and safety profiles [242].

Based on the data at hand, the incorporation of nutraceuticals with recognized antihypertensive efficacy in humans, alongside a consistent enhancement of lifestyle and dietary habits, may serve as an effective strategy for managing prehypertensive individuals along with a valuable adjunct to medical therapy for hypertensive patients. Specifically, these include heightened consumption of potassium, calcium, magnesium, fish oil, fiber, nitric oxide donors, naturally occurring antioxidants, and dairy products. Plant-based protein may enhance blood pressure regulation in a substantial population. Nonetheless, there is a necessity for evidence about security on an ongoing basis, especially when used at an increased dosage or in combination. Additional medical studies are required to discover the active nutraceuticals that possess the ideal cost-effectiveness and hazard–benefit ratio for extensive application in the wider demographic based on the lowest heart disease risk linked to uncomplicated hypertension [243].

Quercetin is the most widespread and extensively studied flavonol associated with cardioprotective properties and a decreased likelihood of vascular disorders. Research highlights that quercetin reduces systolic BP in normotensive individuals and diastolic BP in (pre)hypertensive individuals, suggesting its potential efficacy in achieving this goal. Nevertheless, further research on this subject is required, especially with hypertensive individuals and the consequences of prolonged treatment [244]. Research on experimental animals indicates a beneficial effect of isorhamnetin consumption in the therapy of HTN [245]. One study demonstrated the correlation between prolonged dietary isorhamnetin intake and SBP levels in male individuals. The link could not be determined regarding additional flavonols or for DBP [246]. The preventive beneficial impact of cocoa on BP is attributed to flavanols present in cocoa as monomers (epicatechin and catechin), which are believed to enhance the bioavailability of nitric oxide, hence stimulating vasodilation. Although the BP-lowering impact of epicatechin consumption is limited to those with prehypertension and HTN, it is essential to recognize that these constitute the principal populations who may gain from this intervention for both prevention and management of HTN [247]. A recent study concluded that a two-week oral administration of epicatechin to young borderline hypertensive rats resulted in a sustained reduction in BP for a two-week period following the termination of therapy [248].

## 4. Conclusions

The growing prevalence of hypertension and its related comorbidities has prompted the need to develop new therapeutic agents, as the existing ones, among their effectiveness in reducing BP, are accompanied by complications during long-term administration. In the near future, these novel treatments may be revealed to be more successful and safer than the present means of treating hypertension, since extensive research is necessary to thoroughly investigate the therapeutic properties of these medications.
